# The Intersexual Genetic Correlation for Lifetime Fitness in the Wild and Its Implications for Sexual Selection

**DOI:** 10.1371/journal.pone.0000744

**Published:** 2007-08-15

**Authors:** Jon E. Brommer, Mark Kirkpatrick, Anna Qvarnström, Lars Gustafsson

**Affiliations:** 1 Department of Biological and Environmental Sciences, University of Helsinki, Helsinki, Finland; 2 Section of Integrative Biology, The University of Texas at Austin, Austin, United States of America; 3 Department of Ecology and Evolution, Uppsala University, Uppsala, Sweden; Queens University, Canada

## Abstract

**Background:**

The genetic benefits of mate choice are limited by the degree to which male and female fitness are genetically correlated. If the intersexual correlation for fitness is small or negative, choosing a highly fit mate does not necessarily result in high fitness offspring.

**Methodology/Principal Finding:**

Using an animal-model approach on data from a pedigreed population of over 7,000 collared flycatchers (*Ficedula albicollis*), we estimate the intersexual genetic correlation in Lifetime Reproductive Success (LRS) in a natural population to be negative in sign (−0.85±0.6). Simulations show this estimate to be robust in sign to the effects of extra-pair parentage. The genetic benefits in this population are further limited by a low level of genetic variation for fitness in males.

**Conclusions/Significance:**

The potential for indirect sexual selection is nullified by sexual antagonistic fitness effects in this natural population. Our findings and the scarce evidence from other studies suggest that the intersexual genetic correlation for lifetime fitness may be very low in nature. We argue that this form of conflict can, in general, both constrain and maintain sexual selection, depending on the sex-specific additive genetic variances in lifetime fitness.

## Introduction

In a sexual population, the expected change in mean fitness across generations is given by the additive genetic variance in lifetime fitness [1], and other traits will evolve as a correlated response to this change [2]. The additive genetic variance in fitness is thus an important quantity in evolutionary biology, because without such variance individuals belonging to future generations will not become better adapted to the environment as compared to their ancestors. However, quantitative genetic models rarely take into account that males and females may have different additive genetic variance for fitness, despite the fact that the few studies that have calculated sex-specific additive genetic coefficients of variation for lifetime fitness in wild populations suggest there are differences between the sexes in this respect (Table 1). Further, genes that perform well in males may not do well in females, and vice versa. Recent work has shown that fitness genes may often be sexually antagonistic, with positive effects in one sex, but neutral or even harmful effects in the other sex [3]–[7]. Sexual antagonism and other mechanisms can cause the genetic correlation between male and female fitness to be small or even negative [8], [9].

**Table 1:** Published estimates of additive genetic variance in estimates of lifetime fitness in wild populations.

Organism	Method	Sex	*n*	mean	V_A_ (s.e.)	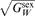	Reference
Collared flycatcher	PO	Male	652	2.35	0.160 ^n.s ^ †	0.17	[24]
(*Ficedula albicollis*)		Female	719	2.21	0.406 ^**^ †	0.29	
Red deer	AM	Male	284	0.98	0.61 (0.59) ^n.s.^	0.80	[9]
(*Cervus elaphus*)		Female	301	2.27	0.99 (0.62) ^n.s.^	0.44	
Great tit	AM	Male	1,631	1.108	0.031 (0.072) ^n.s.^	0.16	[25]
(*Parus major*)		Female	1,795	1.113	0.004 (0.078) ^n.s.^	0.057	

Lifetime fitness was estimated as the sum of offspring recruited into the breeding population (Lifetime Reproductive Success). The method of calculating the variance was based either on parent-offspring regression (PO) or on an animal model (AM). Sample sizes are denoted by *n*. For each organism and each sex, we report the mean, the additive genetic variance components with (between brackets) the standard error and its significance (non significant (n.s.) or P<0.01 (**)) and the sex-specific coefficient of additive genetic variation in LRS (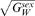 = √(V_A_)/mean).

† Variance component and its standard error not reported by authors; variance component calculated as product of heritability and phenotypic variance.

In this paper, we explore how the intersexual genetic correlation for fitness affects the potential for indirect sexual selection. We begin by reviewing a model that specifies that the critical parameters for estimating the force of indirect selection on mating preferences from empirical data are the sex-specific additive genetic variances in lifetime reproductive success (LRS) and its genetic correlation between the sexes. We then estimate these quantities in the collared flycatcher *Ficedula albicollis*. We have previously [10] shown that a component of fitness is positively correlated across the sexes, but we here explicitly consider lifetime fitness and incorporate the effects extra-pair paternity has on the genetic estimates. Our results indicate that the genetic correlation between male and female lifetime fitness is not large and positive, as is typically implicitly assumed, but is low and negative in sign. We argue that this is consistent with the general picture that is currently emerging, and we discuss the consequences such an intersexual genetic correlation may have for our understanding of sexual selection.

Mating preferences may evolve through indirect sexual selection by a number of potential processes. In a Fisher–Zahavi process [11], males display a heritable secondary sexual trait (ornament) that acts as a sexual signal, and females have a heritable preference for such ornaments. The ornament is either attractive *per se* [1] or carries a cost (handicap) and therefore shows the individual's ability to function despite this handicap [12]. Hence, by mating with a highly ornamented male, a female assures that her offspring inherits from their father the genes that allow him to function despite the ornament's handicap. In a good-genes process, a female chooses a male for his positive genetic contribution to her offspring's fitness. The latter process includes more varied pathways by which positive genetic effects on fitness can be mediated, but the two scenarios are not mutually exclusive [11], [13] and both of them imply–from a quantitative genetic point of view–an additive genetic correlation between male ornament and lifetime fitness [14], [15]. That is, the breeding value (i.e. expected trait value based solely on the genes for that trait) of a male ornament needs to be positively correlated with his breeding value for lifetime fitness, irrespective of the envisioned model determining fitness.

Kirkpatrick and Hall [16] derived a theoretical expression for the change in mating preferences due to indirect sexual selection that makes quite general assumptions regarding genetics and behavior. The results apply to any mating system, any type of mate choice behavior, and any number of genes affecting the female preference, male ornament, and lifetime fitness. The main restrictions are that genes have mainly additive action, that linkage between them not be too tight, and that individual alleles not have very large fitness effects. Here we assume that the loci contributing to genetic variation in lifetime fitness are autosomal, but it is possible to allow for sex-linked genes as well. Results derived by [16] show that the potential for indirect selection on (genetic benefits for) a female mating preference depend on three factors. The first is the accuracy with which females choose males that have high fitness genotypes. The second is the amount of genetic variation for lifetime fitness in males and females. The third factor is the degree to which a genotype that produces high fitness in males also produces high fitness when expressed in females. We here focus on this latter aspect.

The force of indirect selection can be expressed by the number of phenotypic standard deviations that it causes mean preference to evolve each generation (Δ*_I_*). Minor modifications to Equation (2) in ref. [16] give
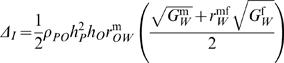
(1)The first parameter on the right, *ρ_P0_*, is the phenotypic correlation among mated pairs between the female's preference and the male's ornament. The next two parameters, *h* ^2^ *_P_* and *h_0_*, are respectively the heritability of the preference and the square root of the heritability of the ornament. The quantity *r* ^m^ *_OW_* is the genetic correlation between the male ornament and male lifetime fitness. Together this first group of terms reflects the accuracy with which female preference genes become associated with genes that produce high fitness in males. Because this first group of terms are correlations and heritabilities, with a maximum of one, the bracketed expression of equation (1) sets the maximal potential change in mate preference. Inside the parentheses of equation (1) are two terms corresponding to the indirect selection on preference genes produced by selection on lifetime fitness in males and in females. The quantities 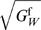 and 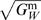 are the additive genetic coefficients of variation for lifetime fitness in females and males, respectively. The quantity *r* ^mf^ *_W_* is the genetic correlation between lifetime fitness in males and females.

This equation draws attention to two key empirical questions. The first concerns the magnitudes of the genetic variances for male fitness and female fitness. The second is the degree to which a genotype that produces high fitness in males will also give high fitness when expressed in females. If the correlation *r* ^mf^ *_W_* is large and positive, for example, then females mating with high fitness males can expect on average to have high fitness sons and daughters. On the other hand, if the genetic correlation between male and female fitness is negative, then a female who mates with a high fitness male will on average have high fitness sons but low fitness daughters. This can diminish or even eliminate the potential for indirect genetic benefits to mate choice.

## Results

Heritability (*h* ^2^±s.e.) of Lifetime Reproductive Success (LRS) was 0.027±0.014 and 0.016±0.018 for females and males respectively. The additive genetic variance of males was low, and the estimated coefficient of additive genetic variation in females was therefore almost double the males' coefficient (Table 2), indicating that the potential for evolution in this population may primarily be determined in females. Our results further show that the estimated genetic correlation in LRS between male and female collared flycatchers was negative, and clearly significantly lower than unity, as judged by a likelihood ratio test (Table 2). Given the proportionally low amount of additive genetic effects in LRS, especially in males, there were large confidence intervals around the estimate of the genetic correlation. However, our results show that breeding values for lifetime performance do not have the same ranking order in male and female collared flycatchers, and show evidence of antagonistic effects across the sexes. Simple comparison of LRS of offspring to their parents confirmed that sons resembled their father more (−0.0082±0.034, n = 880) than daughters did (−0.044±0.032, n = 950), and daughters resembled their mother more (0.015±0.032, n = 1,034) than sons did (−0.032±0.032, n = 977).

**Table 2:** Descriptive statistics, additive genetic variance components and genetic correlation between the sexes in Lifetime Reproductive Success in the collared flycatcher.

Sex	*n*	mean±s.d	V_A_±s.e.	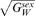	*r^mf^_W_*±s.e.	LRT *r* = 1
Male	3,109	0.92±1.23	0.043±0.051	0.217		
					−0.85±0.59 a	χ^2^_1_ = 6.6, P = 0.01
Female	3,972	0.77±1.16	0.074±0.040	0.353		

The estimate of additive genetic variance V_A_, the genetic correlation between the sexes and their standard errors are derived from an animal model, correcting for differences in the ‘cohorts’ (year of first breeding) and study plots. Test of the significance of estimates are based on a Likelihood Ratio Test (LRT) comparison of the likelihood of the full model with a model where the genetic correlation is specified, based on (a) setting the genetic covariance to zero (LRT *r* = 0), and setting the intersexual genetic correlation to+1 (LRT *r* = 1). The coefficient of additive genetic variation is indicated as 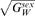.

a LRT *r* = 0: χ^2^_1_ = 2.3, P = 0.13

The low intersexual genetic correlation essentially nullified any possibility for indirect sexual selection to operate, since the weighted average of the male and female coefficient of additive genetic variation [bracketed expression in equation (1)] became very low and slightly negative (0.217−0.85×0.353 = −0.083).

### Sensitivity of results to simulation of extra-pair paternity

**Figure 1:**
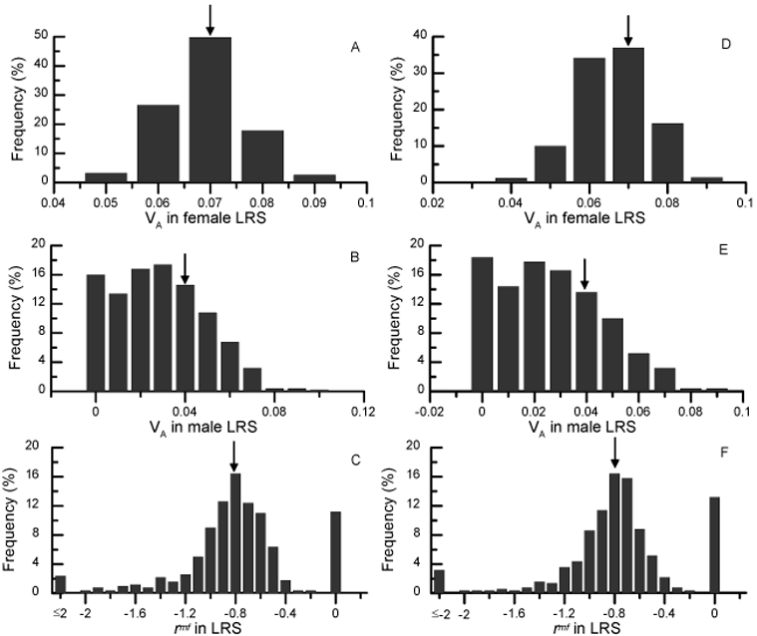
Simulations of results incorporating extra-pair paternities. Simulations either not assigned a random 15% of offspring (left panels, A–C) or directionally assigned them to a local male with a broader forehead patch (right panels, D–F). The frequency distributions of additive genetic variances in female LRS (A, D) and male LRS (B, E), and the intersexual genetic correlation in LRS (C, F) are based on 500 simulations. Correlations were left unconstrained and could therefore be lower than −1. The values based on the social pedigree (Table 2) are indicated with an arrow.

Collared flycatchers engage in extra-pair mating. On average, 15% of offspring have extra-pair paternity (EPP), which introduces errors both in the pedigree and the fitness estimates underlying our analyses. We explored how sensitive our conclusions are for such errors by simulating two EPP scenarios. Our first scenario considered EPP to be random, and in a second simulation scenario paternity of recruits was directionally assigned to contemporary local males that had a broader forehead patch than the social father (see Methods). We carried out 500 simulations where we either omitted or directionally re-assigned paternity information from a randomly chosen 15% of recruits and recalculated male LRS and animal-model estimates. As expected, our estimate of female additive genetic variance was not much affected by simulating EPP, with deviations distributed symmetrically around the original estimate (Fig. 1A). Directional assignment tended to reduce female additive genetic variance somewhat (Fig. 1D). Male additive genetic variances tended to diminish rather than increase (Fig. 1B, 1E), and were zero in 11.2% (56/500) and 13.2% (66/500) of runs in the random and directional assignment models respectively. Overall, the negative sign of the genetic correlation in LRS between the sexes proved to be a robust feature of the system, since none of the simulations indicated that incorporation of EPP could change this correlation to a positive one (Fig 1C, 1F). At the same time, however, this correlation was zero in all those cases when male additive genetic variance was absent, and became lower than −1 in 18.4% (92/500) of random simulations (Fig. 1C), and in 17.2% (86/500) of simulations based on directional assignment (Fig. 1F). Simulations based on directional assignment tended to make the intersexual genetic correlation slightly more positive (mean−0.83) compared to the random model (mean−0.87). Because a correlation is proportional to the inverse of the standard deviations of the underlying variables, reasonable correlations were only found when male additive genetic effects were fairly large (r_s_ between male additive genetic variance and the genetic correlation was 0.16 and 0.39 for random and directional assignment simulations respectively).

Calculating the weighted average of the coefficients of additive genetic variation in LRS [bracketed expression in equation (1)] for each of the models produced an average of 0.033±0.066 (s.d.) for the random model. Because directional assignment tended to reduce the female additive genetic correlation and increase the genetic correlation, the resulting total coefficient became more positive, 0.081±0.11 (s.d.). Nevertheless, these estimates confirm that this quantity is likely to be low. Most importantly, sexually antagonistic effects much reduced the total coefficient of additive genetic variation in LRS compared to the sex-specific coefficients (Table 2).

## Discussion

Sexual selection concerns both sexes, and must acknowledge the evolutionary dynamics of both males and females and any interaction between them. Indirect genetic benefits that come from mate choice depend not only on how genes that a female chooses in her mate are expressed in males (her sons), but also on their effects in females (her daughters). Indirect selection on preferences will be reduced if there is limited genetic variation for fitness in either males or females. Indirect sexual selection will also be diminished if the genetic correlation between male and female lifetime fitness is small or negative. Our results suggest that both of these mitigating circumstances are at work in collared flycatchers. Using over twenty years of individual-based records of collared flycatchers, we estimate the genetic correlation in LRS between the sexes, and find this to be negative in sign (although not significantly different from zero). We further find an indication of a larger sex-specific coefficient of additive genetic variation in female than in male LRS. Together these quantities act to essentially nullify the scope of indirect sexual selection on mate choice in this species.

Our review of theory illustrates how important the sex-specific additive genetic (co)variances are for a proper understanding of evolutionary dynamics. On the other hand, our review of empirical studies and our own results underline that quantification of such (co)variances typically deals with small effect sizes that are not significantly different from zero. Except in female collared flycatchers, lifetime fitness has not been shown to be significantly heritable in the wild (Table 1). Clearly, only small genetic effects are expected for lifetime fitness, because selective processes will have largely eroded these [1]. Statistical techniques that have been developed to estimate quantitative genetic parameters, such as the animal model, calculate an unbiased estimator for the effects that genes have. Despite their low statistical significance, the estimates presented in this and other quantitative genetic studies on lifetime fitness (Table 1) provide our best understanding of processes that are key to understanding evolution in the wild.

We find that the intersexual genetic correlation in LRS in the collared flycatcher is negative in sign (−0.85). This result is in accordance with laboratory studies on *Drosophila melanogaster* and a study on red deer which have both found low intersexual genetic correlations in LRS that are non-significantly different from zero of −0.16, [4] and −0.48 [9] respectively. We find that the intersexual genetic correlation in LRS is significantly below one (see also [9]), indicating that fitness effects of genes expressed in males are not positively correlated to their effects in females. Much of the literature on sexual selection and evolution in general implicitly assumes that genetic fitness effects correspond closely across the sexes. In a sexual population, there are several factors which may cause the intersexual genetic correlation for fitness to be substantially less than 1. Firstly, male and female evolutionary interests need not align, and there has been growing awareness of the existence of sexually antagonistic genetic effects [3]–[7], [9]. Such sexual conflict will strongly reduce the intersexual genetic correlation for fitness [8]. A low intersexual genetic correlation in fitness will have consequences for evolutionary dynamics in general, because selection in one sex will be counteracted by the selection on those genes in the other sex. A proper measure for an evolutionary conflict between the sexes is based on lifetime performance [15], [16]. This is because components of fitness are likely to trade-off against each other, such that any particular component may poorly reflect total fitness. In *Drosophila melanogaster*, the intersexual genetic correlation based on a juvenile fitness component was positive, but changed to a negative correlation for the adult fitness component, thereby nullifying the genetic correlation between the sexes for total fitness [4]. In collared flycatchers, annual fitness (a component of LRS) is positively genetically correlated across the sexes [10], whereas LRS is negatively correlated between sexes (this study). By contrast, morphological traits, which are typically poorly correlated with fitness, have a high (>0.8) genetic correlation across sexes [17], [18]. Possibly, conflict builds up across the trait continuum of morphology to life history, although conclusive evidence for this assertion from a single study system currently is lacking.

Sex-specific gene expression may be a second factor contributing to a low intersexual genetic correlation. Traits that are expressed only in one sex can contribute to genetic variance for fitness in that sex but will decrease the fitness correlation between sexes. Many genes coding for traits involved in reproduction (i.e. traits linked to fitness) may have a sex-specific expression. For example, the seasonal timing of laying has important selective consequences, but is not affected by males in this collared flycatcher population [19]. Females are the heterogametic sex in birds and important life-history traits, which act to enhance fitness, may even be sex-chromosome linked (c.f. *Drosophila melanogaster*, [7]). Sex-specific gene expression and sex linkage can be viewed as adaptations to sexual antagonistic fitness effects, because they will ameliorate their overall fitness consequences.

Our simulations reveal that a negative intersexual genetic correlation is a robust feature of this system, which occurs also when misassignment of paternities is simulated for. The low additive genetic variance in male LRS is clearly the critical aspect of this system when allowing for paternity misassignment. About 15% of collared flycatcher offspring result from extra-pair copulations ([20], L. Gustafsson and H. Ellegren unpubl.) creating errors in the paternity assessment in the pedigree. Typically, the influence of these errors on parameter estimates are simply ignored in animal model analyses in wild avian populations (but see [21]), and their effects on genetic covariances remain largely unexplored. Our simulations either not assign 15% of recruits to any father, or directionally assign them to contemporary males with a broader forehead patch. The former is the most objective way of treating extra-pair matings, since it does not require making any assumptions about the direction for assigning offspring to other males. However, gaining extra-pair paternity is an inherently non-random process in the collared flycatcher [20] which acts to enhance the skew in male LRS in the population. The sensitivity of results to this non-random aspect therefore needs to be taken into account. In the majority of simulated datasets, male additive genetic variances in LRS becomes lower, thereby leading to extreme or zero estimates for the genetic correlation. These findings are likely to be general, because uncertainty in parental assignment is a typical feature in natural populations, either because of extra-pair paternity [22] or because of limits to the reliability of molecular paternity assignment based on a finite set of markers (e.g. [23]). Our simulations reveal that directional assignment of paternities causes a (small) directional shift in the additive genetic (co)variances towards lower estimates of the additive genetic variance in female LRS and a more positive genetic correlation. This illustrates the interrelatedness of the genetics of males and females in a two-sex model. Consequently, the overall coefficient of additive genetic variation is slightly increased to allow a maximal potential shift in mate preferences of, on average, 8% of a standard deviation. Hence, extra-pair copulatory behaviour *per se* has, in this population, the capacity to modestly increase the potential for sexual selection.

### Conclusions

A low intersexual genetic correlation in lifetime fitness is thought to reduce the scope for indirect sexual selection, because males with high fitness will produce average daughters [7], [8], [9]. However, as we have shown here, the scope of indirect sexual selection will not only be a function of the expression of male fitness genes in females (as quantified by the intersexual genetic correlation in lifetime fitness), but also of the sex-specific coefficients of additive genetic variance. A low intersexual genetic correlation in fitness acts to maintain additive genetic variance in fitness because the evolutionary trajectories of the two sexes do not coincide, and thus also has the potential to maintain sexual selection. In particular, strong additive genetic effects of genes for fitness in males will maintain the potential for indirect sexual selection, even if none of these effects are correlated with the effects in females, in case females have a low coefficient of additive genetic variance in fitness. For example, in red deer, male and female annual fitness are significantly negatively genetically correlated (−0.95), but males have a much higher coefficient of additive genetic variance (1.56) compared to females (0.44) [9]. Consequently, sexually antagonistic effects reduce the scope for indirect sexual selection only marginally in this species (maximum rate, as given by the bracketed expression in equation (1), is 1.56−0.95×0.44 = 1.14 SD in mate preference per year). Red deer have a mating system where most of the paternity in a given year goes to one male (lekking), and a high coefficient of additive genetic variance in male red deer fitness is thus expected. On the other hand, our empirical results on collared flycatchers show that a low intersexual genetic correlation in fitness acts mostly to constrain sexual selection in this largely monogamous passerine. A comparison across species with various mating systems will thus be highly instructive. Our results do, however, highlight that the main challenges for modeling intersexual genetic relationships in lifetime fitness in the wild are the low sex-specific additive genetic variances in fitness, in combination with the incorporation of uncertainty in paternity.

## Methods

### Study species and calculation of lifetime fitness

Collared flycatchers were studied on the island of Gotland in the Swedish Baltic sea from 1980 and onwards. These birds breed in nest boxes that were supplied in ample numbers in a series of forest patches (plots). Individuals were ringed either as nestlings or when trapped at the nest as adults in order to allow lifelong individual identification and assessment of yearly reproductive success. Lifetime fitness was estimated as Lifetime Reproductive Success (LRS), the sum of all recruits (offspring that recruited back into the breeding population) of both sexes produced during an individual's lifetime. We here only used data on individuals that bred in the core patches of the study area which have been intensively monitored, and which started to breed in 1981–1999 in order to collect lifetime data on recruitment. Parts of the population have been involved in life-history experiments where a component of their reproductive output was manipulated. However, exclusion of such data necessarily leads to exclusion of the complete records of an individual, and could produce a bias in the animals used to estimate LRS versus the complete population, because such experiments are typically performed on ‘average’ individuals. Furthermore, we wanted to maximise the sample size and hence the power in our calculations of, especially, the genetic correlation in lifetime fitness across the sexes. We therefore considered the sum of all recruits that were raised in an individual's nest (independently of their origin) summed up for all individuals, irrespective of whether the individual was involved in an experiment at one point during its lifetime.

### Estimating genetic (co)variances in Lifetime Reproductive Success

We partitioned the variance in LRS using a REML linear mixed model that incorporates pedigree-based estimated of genetic relatedness across all individuals (animal model, [17], [23]), which was implemented in AsReml (VSN International). We included as fixed effects the year an individual bred for the first time in order to correct for differences in LRS across ‘cohorts’ experiencing different environmental conditions during their lifetime, and the study plot in which the individual bred. As random effects, we estimated the additive genetic variances for males and females and the covariance between them. The estimation of the additive genetic (co)variances was based on pedigree information of 3,557 individuals that recruited back into the breeding population and for whom at least one parent was known. All other individuals were considered as base individuals. The animal model estimates the additive genetic covariance across sexes as a function of the covariance between opposite-sex relatives. Residual errors were set to be uncorrelated (i.e. zero) across sexes, because these cannot be estimated since sex-specific traits are not measured on the same individual.

We tested for the significance of the additive genetic covariance by a Likelihood Ratio Test (LRT) based on −2×difference in log-likelihood between the unconstrained model specified above and a model with the intersexual genetic correlation set to zero, which has one degree of freedom. In addition, we specifically tested whether the confidence intervals around the genetic correlation differed from 1, by performing a LRT between the full model and a model where the genetic correlation was set at unity.

The direction of the animal-model's genetic correlation was verified by calculating the resemblance of the LRS of male and female offspring to the LRS of their male and female parent. Prior to each analysis, LRS values were corrected for differences between cohort years and areas, and standardized to zero mean and unit standard deviation. Values of multiple same-sex offspring were averaged. Resemblance was calculated as the slope of a linear regression between sex-specific offspring and parent values.

### Accounting for extra-pair paternities in the pedigree

There will be misassigned paternal links in the collared flycatcher pedigree, because about 15% of offspring are not sired by their social father, i.e. the male that provides care for them during the nestling phase [20]. Such errors have been shown to have relatively minor effect on the estimation of animal-model derived variances for morphological traits [21]. However, a systematic investigation of the consequences of pedigree errors for the accuracy of genetic correlations has not been made. Furthermore, in terms of LRS, extra-pair paternities will not only affect the pedigree links, but also the trait values for the males, since each cuckolded recruit will reduce the social male's LRS, but increase the extra-pair father's LRS. Hence, simulation results based on morphology cannot necessarily be applied to estimates of individual fitness such as LRS.

We carried out two different simulations. In the first simulation, we assumed that for a randomly chosen 15% of recruits paternity was misassigned, and set their fathers as ‘unknown’. We then recalculated the LRS of all males based on this simulated pedigree. Some males' LRS was thus reduced. In this approach, 15% fewer recruits were assigned to a male parent, thereby reducing the amount of information in the pedigree and thus the power of the animal model in describing additive genetic (co)variances. Our second approach recognized that social males were cuckolded by males with a broader forehead patch [20]. Consequently, extra-pair paternities (EPP) will affect the estimated LRS of males non-randomly. For a randomly chosen 15% of recruits, paternity was assigned to a local male (i.e. a male breeding in the same year in the same study plot) with a broader forehead patch than the social male. We assigned paternity to a random local male in case such assignment was not possible, either because the social male's forehead patch was not measured or because the social male's forehead patch was the broadest forehead patch of all local males. Hence, in this simulation, some males' LRS was reduced whereas others' was increased, and in this simulation approach the same number of recruits were assigned to fathers as in the social pedigree thereby keeping the amount of information approximately equal. Again, the calculations for LRS were adjusted for the simulated pedigree.

We simulated LRS data and pedigree 500 times, and calculated the additive genetic variances of males and females and the intersexual genetic correlation for each of the simulations. The additive genetic covariance matrix was left unconstrained, because constraining this matrix to be general positive led to failure of model convergence in some iteration (see results). Correlations could therefore exceed the range of −1 to 1. Conditional on our assumptions, these distributions will be informative of the robustness of our estimates based on the social pedigree with respect to extra-pair paternities, and we compared our observed values with the simulated values in order to assess the direction and possible bias of extra-pair paternities. The simulation of the pedigree and recalculation of LRS data was implemented with a purpose-specific programme coded in MATLAB (MathWorks), with animal model analyses implemented in AsReml (VSN International) as described above.
